# Reduction in inequalities in health insurance coverage and healthcare utilization among older adults in the Philippines after mandatory national health insurance coverage: trend analysis for 2003–2017

**DOI:** 10.1186/s12199-020-00854-9

**Published:** 2020-06-09

**Authors:** Kathryn Lizbeth Lucena Siongco, Keiko Nakamura, Kaoruko Seino

**Affiliations:** Department of Global Health Entrepreneurship, Division of Public Health, Graduate School of Medical and Dental Sciences, Tokyo Medical and Dental University, Yushima 1-5-45, Bunkyo-ku, Tokyo, 113-8519 Japan

**Keywords:** National Health Insurance Program, Healthcare utilization, Health policy, Inequalities, Older adults, Concentration index, Philippines

## Abstract

**Background:**

Health policies in the Philippines have evolved in response to increasing health demands of older adults. However, there is a lack of research on equity among the ageing population in low-middle income countries. The objective of this study was to identify the trends in National Health Insurance Program (NHIP) coverage and healthcare utilization among older adults in the Philippines for the period from 2003 to 2017, during which NHIP expansion policies were implemented, focusing on reductions in socio-economic inequalities.

**Methods:**

A literature search of policies for older adults and an analysis of four Philippine National Demographic and Health Surveys (2003, 2008, 2013, and 2017) with data from 25,217 older adults who were 60 years or older were performed. The major outcome variables were NHIP coverage, self-reported illness, outpatient healthcare utilization, and inpatient healthcare utilization. Inequalities in NHIP coverage and healthcare utilization according to wealth were evaluated by calculating the concentration index for individual years, followed by a regression-based decomposition analysis.

**Results:**

NHIP coverage among older adults increased from 9.4 (2003) to 87.6% (2017). Although inequalities according to wealth quintile were observed in all four surveys (all *P* < 0.001), the concentration index declined from 0.3000 (2003) to 0.0247 (2017), showing reduced inequalities in NHIP coverage over time as observed for self-reported illness and healthcare utilization. NHIP coverage expansion for older adults in 2014 enabled equal opportunity for access to healthcare.

**Conclusion:**

The passage of mandatory NHIP coverage for older Filipino adults in 2014 was followed by a reduction in inequality in NHIP coverage and healthcare utilization according to wealth.

## Background

The Philippines is considered to have an ageing population, similar to neighboring low- and middle-income countries (LMICs). The number of older adults (individuals aged 60 years or older) in the Philippines was reported be 8 million in 2017 and is estimated to increase to 21 million in 2050, accounting for 14% of the total population [1]. Given the growth of the older population, the demand for health services is increasing because of complex healthcare needs. In response to the increasing health demands of older adults, widespread efforts to improve health service delivery have been prominent in the Philippines as part of efforts to overcome socio-economic disparities in accessibility and availability of resources. Promoting fair financing and better health access across LMICs is necessary, as equitable access to healthcare services is a major public health challenge.

Social health insurance is a form of financing strategy when working towards equitable healthcare financing. The passage of Republic Act 7875 in 1995 created the Philippine Health Insurance Corporation (PhilHealth) and established the National Health Insurance Program (NHIP) with four initial membership categories: (i) formal sector, (ii) indigents, (iii) retirees, and (iv) individual. Through the years, the Department of Health (DOH) has provided a budget for PhilHealth to cover the contributions for indigent members. In 2012, the introduction of a “Sin Tax” Law, which is an excise tax on alcohol and tobacco products, allocated 85% of its revenues to healthcare [2], raising more than 1.2 billion USD (US dollars) in its first year and allowing the enrollment of 45 million Filipinos into PhilHealth [3]. Furthermore, as a result of the amendment of the “Expanded Senior Citizens Act of 2010” in 2014, individuals aged 60 years or older were automatically enrolled into the NHIP and given free inpatient healthcare at government hospitals, thereby including “senior citizens” as a sector receiving NHIP coverage [4]. However, the urge to speed the expansion of coverage and the provision of free healthcare services to older adults holds the potential to impose a heavy financial burden on the Philippine health system, as previously experienced in Japan [5].

The Philippines’ rapid economic growth has enabled advancements in healthcare in recent years; however, the health system remains fragmented. Large disparities in access to healthcare services remain, with differences observed among socio-economic groups, geographical regions, and rural/urban residences [6]. In a report on equity in health and healthcare in the Philippines, the poor were shown to suffer a greater burden of diseases [7], with high inequity regarding health outcomes between socio-economic groups [6]; these data suggest that to promote vertical equity, healthcare must be concentrated among the poor, who have greater healthcare needs. However, the poor are unable to acquire necessary treatment according to their needs. All individuals should gain equitable access to healthcare in relation to need to achieve universal health coverage (UHC). Healthcare need is partly proxied by self-reported illness across socio-economic groups, which is reported as an illness or symptom in the previous 30 days in household surveys [8]. In 2011, the Philippine government launched a UHC strategy to improve the overall health system and provide the poor with protection from financial risks [9]. To attain health equity, efforts must be directed in assessing the inequality in health and use of healthcare services of the vulnerable population [10], and socio-economic inequalities are reported through the prevalence of morbidities [11] and access to healthcare services [12, 13]. The implementation of the NHIP and the expansion of coverage to include the older adult population are expected to reduce health inequalities through the provision of quality healthcare services and improved access to healthcare.

Despite the implementation of policies to increase NHIP coverage and access to healthcare, there remains a lack of research on equity among older adult populations in LMICs. This paper is the first to evaluate socio-economic inequalities in health and healthcare utilization among older adults in the Philippines following the expansion of NHIP coverage as a step towards achieving UHC. We used population-based surveys from 2003 to 2017 to identify existing socio-economic inequalities in health and healthcare utilization among older adults in the Philippines. The objectives of this study were therefore to analyze the trends in NHIP coverage and healthcare utilization among older adults in the Philippines from 2003 to 2017, during which period the NHIP expansion policies were implemented, and to analyze the reduction in socio-economic inequalities, and present key developments in policies benefitting older adults in the Philippines in relation to NHIP coverage expansion and healthcare use.

## Methods

This study consists of a literature search and an analysis of four Philippine National Demographic and Health Surveys conducted in 2003, 2008, 2013, and 2017.

### Literature search

A literature search including data from PhilHealth, DOH, and technical reports from national organizations (a total of 25 files) was performed to identify key developments in health policies benefitting older adults in the Philippines, even before the conception of the National Health Insurance Act in 1995. The chronological arrangement of policies presented in Table 1 shows the developments in relation to NHIP coverage and healthcare utilization among older Filipino adults.

**Table 1:** Developments in health policies and laws concerning older adults in the Philippines

Year	Title/description
1992	RA 7432; an act to maximize the contribution of senior citizens to nation building, grand benefits and special privileges, and for other purposes
1995	RA 7875; National Health Insurance Act of 1995—creation of PhilHealth with coverage under 50%
2004	RA 9257; Expanded Senior Citizens Act of 2003, an act granting additional benefits and privileges to senior citizens and amending RA 7432
2004	RA 9241; an act amending RA 7875, stipulated that indigent contributions shall be subsidized by the local government units (LGUs) and the national government
2004	Executive Order 276 s. 2004; “Plan 5/25M” enrolled 4.2 million poor families or 21.2 million individuals into the PhilHealth program, funded by the Department of Budget and Management (DMBM) and Philippine Charity Sweepstakes Office (PCSO)
2004	RA 9334; the Congress, through this act, earmarked 2.5% of government revenues from taxes on sin products to the National Health Insurance Program (NHIP)
2008	RA 9502; Universally Accessible Cheaper and Quality Medicines Act providing for cheaper and quality medicines and amending other related laws
2010	RA 9994; Expanded Senior Citizens’ Act of 2010, stipulating that all indigent senior citizens shall be covered by the NHIP of PhilHealth
2010	Department of Health (DOH) Administrative Order (AO) 2010-0036; focused on increasing enrollment coverage, improving availment of benefits and improved facility preparedness through the Health Facility Enhancement Program (HFEP)
2011	PhilHealth Circular No. 011-2011; “No Balance Billing Policy” applied to all PhilHealth Sponsored Program members for specified cases
2012	PhilHealth Circular No. 048-2012; the Z Benefit Package developed by PhilHealth aimed to increase financial risk protection using cost-effective interventions
2012	RA 10351; Sin Tax Law—restructuring the excise tax on alcohol and tobacco products and amending the National Internal Revenue code of 1997
2013	RA 10606; amending RA 7875, shifted subsidizing PhilHealth premiums for Sponsored Program families to the DOH, and the Sin Tax Law to finance expanding of the Sponsored Program
2014	RA 10645; an act providing for the mandatory PhilHealth coverage for all senior citizens and amending the Expanded Senior Citizens Act of 2010
2019	RA 11223; Universal Health Care (UHC) Act prescribing reforms in the healthcare system

### Analysis of Philippine National Demographic and Health Survey data

The analyses were based on representative, cross-sectional Philippine National Demographic and Health Surveys (PDHS) conducted in 2003, 2008, 2013, and 2017 [14–17]. The following numbers of individuals aged 60 years and older were included in each survey: 3921 (2003), 4393 (2008), 5571 (2013), and 11,402 (2017). Cases with missing data for at least one study variable were excluded from the analysis (2003, 22 [0.6%]; 2008, 8 [0.2%]; 2013, 7 [0.1%]; 2017, 33 [0.3%]). The final set of data for each survey used in the analysis included 3899 (2003), 4385 (2008), 5564 (2013), and 11,369 (2017) older adults.

### Variables

The outcome variables of this study included National Health Insurance Program coverage, self-reported illness, and healthcare utilization, which were composed of outpatient care utilization and inpatient care utilization. Independent variables included socio-economic characteristics such as age, gender, place of residence, educational attainment, wealth, number of household members, and relationship to household head.

#### National Health Insurance Program (NHIP) coverage

The outcome variable “health insurance” was measured by creating a binary variable for those with NHIP membership or PhilHealth, regardless of the category of membership (formal or informal, indigent, sponsored, lifetime, senior citizen, or overseas).

#### Self-reported illness

The outcome variable “self-reported illness” was composed of individuals who reported having at least one illness (non-communicable disease [NCD], communicable disease [CD], or injury) within the last 30 days prior to the interview. The variable was dichotomized as no = 0 or yes = 1. Individuals who reported having at least one illness in the last 30 days were categorized as “yes”; otherwise, the individuals were categorized as “no.” Self-reported illness was excluded from the 2003 data because of a lack of information.

#### Healthcare utilization (outpatient care utilization and inpatient care utilization)

The measurement of healthcare utilization involved the following questions: for outpatient care, “Where was consultation/advice or treatment first sought for (name)’s illness/injury/check-up/laboratory tests in the last 30 days?”; and for inpatient care, “Where was (name) last confined in the last 12 months?” Those who responded with any public or private sector facility were coded as “care utilization (yes)” as performed in previous studies [7, 18]. In contrast, those who responded with alternative medicine use, non-medical sectors, and others were coded as “non-utilization (no).” An analysis of outpatient care and inpatient care utilization was not performed for the 2003 data because of a lack of information. Each outcome variable was dichotomized as no = 0 or yes = 1.

#### Socio-economic variables

This study included individual and household characteristics that have been theoretically and empirically linked to health insurance ownership [19–21] and health and healthcare utilization outcomes [19, 20, 22].

Each individual’s age was categorized as follows: 60–69 years, 70–79 years, and 80 years or older. Gender was categorized as male or female. Place of residence was categorized as rural or urban. Education was categorized as no education, primary, secondary, or higher. Wealth was based on the computation of the DHS program per the specific survey year. Households were given scores derived using a principal component analysis based on the number and kinds of consumer goods they own. The wealth quintiles were compiled by assigning a household score and ranking to each household member and then dividing the distribution into five equal categories, each compromising 20% of the population. Relationship to the household head was categorized as self, spouse, or others. Number of household members was categorized as 1–2, 3–4, or 5 or more members. Marital status and occupation were not included because of a lack of information.

### Statistical analysis

Descriptive statistics were initially used to analyze the outcome variables according to socio-economic characteristics for each of the four surveys. Chi-square test was used to measure the associations of NHIP coverage, self-reported illness, outpatient care utilization, and inpatient care utilization with older adult characteristics (Table 2).

**Table 2:** NHIP^a^ coverage, self-reported illness, and healthcare utilization^b^ according to characteristics of older adults, Philippines (2003–2017)

Variables	2003 (%), *N* = 3899	2008 (%), *N* = 4385				2013 (%), *N* = 5564				2017 (%), *N* = 11,369			
	NHIP^a^	NHIP^a^	Self-reported illness	Outpatient use	Inpatient use	NHIP^a^	Self-reported illness	Outpatient use	Inpatient use	NHIP^a^	Self-reported illness	Outpatient use	Inpatient use
Overall (%)	9.4	35.3	20.5	13.3	8.8	57.9	27.0	15.6	8.6	87.6	28.3	18.7	12.4
Age (years)													
60–69	10.8	37.2	18.5	12.0	7.0	60.6	23.5	13.0	7.2	85.4	28.6	18.2	11.9
70–79	7.7	33.7	23.5	15.3	10.8	56.7	32.3	20.6	9.6	90.8	29.4	20.2	14.0
80 or above	5.8	29.6	23.0	14.8	12.8	46.4	32.5	16.6	13.1	91.1	23.4	17.4	11.5
	***	**	***	*	***	***	***	***	***	***	***	*	**
Gender													
Male	12.2	36.4	19.6	13.0	9.5	60.7	26.5	13.9	8.3	87.1	36.2	24.9	18.3
Female	6.9	34.4	21.2	13.5	8.2	55.8	27.4	16.9	8.7	88.0	21.9	13.7	7.7
	***					***		**			***	***	***
Residence													
Rural	6.8	32.0	22.8	13.1	9.0	57.2	29.9	16.3	8.3	87.0	29.5	18.7	13.3
Urban	12.7	39.7	17.4	13.5	8.5	59.1	22.5	14.4	9.0	89.1	25.5	18.5	10.4
	***	***	***				***			**	***		***
Education													
No education	2.9	15.3	21.4	10.3	6.3	34.5	28.3	13.2	7.6	74.1	22.7	10.5	8.4
Primary	5.8	28.8	20.9	12.7	8.4	53.5	28.3	15.2	8.6	86.1	29.9	18.8	12.3
Secondary	12.6	42.1	21.5	14.4	10.3	57.9	25.8	15.1	8.1	87.9	29.0	19.6	14.5
Higher	29.3	61.4	17.2	15.5	9.5	79.1	24.5	18.0	9.3	95.3	24.7	19.6	11.6
	***	***				***				***	***	***	***
Wealth index													
Poorest	4.1	20.1	24.2	10.4	6.6	48.3	29.4	12.1	6.1	81.5	29.4	16.3	10.2
Poorer	5.0	28.7	23.6	12.0	8.8	52.4	27.9	15.7	7.3	86.3	30.3	18.4	12.7
Middle	8.4	32.3	20.8	13.5	8.7	55.2	29.0	16.2	10.7	87.4	28.8	19.8	13.8
Richer	11.0	43.5	17.9	14.6	9.9	57.5	25.7	16.5	8.7	89.4	27.7	19.0	13.8
Richest	18.5	50.7	16.1	15.7	9.6	72.3	24.0	16.5	9.4	94.4	24.6	20.2	12.0
	***	***	***	*		***	*	*	**	***	***	**	**
Relationship to HH^c^													
Self	12.2	36.3	22.4	14.9	9.4	60.3	29.7	17.1	8.5	88.3	39.5	28.2	19.8
Spouse	7.4	41.4	22.6	13.8	8.6	65.7	27.1	16.8	8.8	89.1	16.8	6.6	2.0
Others	3.5	26.3	12.9	8.2	7.1	42.2	18.8	9.6	8.5	82.6	2.9	0.6	0.4
	***	***	***	***		***	***	***		***	***	***	***
Number of HH^c^													
1–2	8.7	28.8	28.3	16.4	9.5	52.4	39.6	21.7	8.8	87.5	28.1	17.5	10.6
3–4	10.3	39.3	23.2	14.5	8.3	60.1	27.3	16.9	8.9	88.9	27.4	18.4	11.7
5 or more	9.1	35.8	15.1	11.1	8.7	59.1	20.8	11.7	8.2	86.6	29.1	19.7	14.4
		***	***	***		***	***	***		**			***
NHIP^a^ coverage													
No			19.9	11.7	6.7		25.1	13.0	5.7		22.7	12.7	6.3
Yes			21.5	16.2	12.6		28.5	17.4	10.7		29.1	19.5	13.3
				***	***		**	***	***		***	***	***
Self-reported illness													
No		34.9		5.2	6.9	56.8		6.2	5.9	86.6		8.7	8.4
Yes		37.0		44.8	16.2	61.0		40.7	15.8	90.1		43.9	22.8
				***	***	**		***	***	***		***	***
Outpatient care use													
No		34.1	13.0		6.1	56.6	19.0		6.0	86.7	19.5		8.4
Yes		43.0	69.1		26.5	64.8	70.6		22.3	91.6	66.5		30.0
		***	***		***	***	***		***	***	***		***
Inpatient care use													
No		33.8	18.8	10.7		56.6	24.9	13.2		86.7	24.9	14.9	
Yes		50.8	37.8	40.1		72.1	50.0	40.6		93.8	51.7	44.9	
		***	***	***		***	***	***		***	***	***	

Self-reported illness, outpatient care utilization, and inpatient care utilization data were unavailable in 2003

^*^P < 0.05; ^**^P < 0.01; ^***^P < 0.001

^a^National Health Insurance Program

^b^Outpatient care utilization and inpatient care utilization

^c^Household

#### Concentration index

The concentration index (*C*) was estimated to quantify relative socio-economic inequalities in the NHIP coverage, self-reported illness, outpatient care utilization, and inpatient care utilization outcome variables (Table 3). *C* represents twice the area between the concentration curve and the line of equality (the 45° line), bounded between − 1 and + 1 [23]. A *C* of 0 denotes no socio-economic-related inequality. When the curve lies above (below) the line of inequality, the index acquires a negative (positive) value, therefore indicating a disproportionate concentration of the health or healthcare utilization variable among the poor (rich). For example, a negative *C* for self-reported illness denotes a higher value among the poor.

**Table 3:** Decomposition analysis of inequalities in NHIP^a^ coverage, self-reported illness, and healthcare utilization^b^ among older adults in the Philippines, 2003–2017

Variables	Concentration index (*C*)				Absolute contribution to *C*^c^				Percentage contribution to *C*^c^ (%)			
	2003	2008	2013	2017	2003	2008	2013	2017	2003	2008	2013	2017
NHIP^a^ coverage												
Concentration index	0.3000	0.1803	0.0806	0.0247								
Education												
Primary	− 0.123	− 0.187	− 0.196	− 0.228	− 0.010	− 0.029	− 0.022	− 0.015	− 3.3	− 15.8	− 27.3	− 60.0
Secondary	0.264	0.228	0.143	0.105	0.024	0.023	0.008	0.004	8.0	12.8	9.6	14.6
Higher	0.602	0.564	0.525	0.530	0.164	0.091	0.054	0.024	54.8	50.7	66.8	98.6
Wealth index												
Poorer	− 0.455	− 0.486	− 0.526	− 0.485	− 0.007	− 0.018	0.000	− 0.003	− 2.3	− 10.2	− 0.4	− 11.0
Middle	− 0.054	− 0.106	− 0.137	− 0.114	− 0.004	− 0.005	− 0.001	− 0.001	− 1.4	− 2.5	− 1.1	− 3.1
Richer	0.370	0.314	0.277	0.294	0.027	0.032	0.004	0.003	9.0	17.6	4.9	10.8
Richest	0.793	0.771	0.746	0.755	0.118	0.088	0.044	0.016	39.3	48.7	54.6	64.7
Self-reported illness												
Concentration index		− 0.0972	− 0.0474	− 0.0965								
Education												
Primary		− 0.187	− 0.196	− 0.228		− 0.002	0.009	− 0.004		2.1	− 19.3	4.2
Secondary		0.228	0.143	0.105		0.009	− 0.005	0.000		− 9.6	9.6	− 0.1
Higher		0.564	0.525	0.530		0.002	− 0.021	− 0.023		− 1.7	43.8	24.4
Wealth index												
Poorer		− 0.486	− 0.526	− 0.485		0.000	0.004	0.004		0.2	− 8.9	− 3.9
Middle		− 0.106	− 0.137	− 0.114		0.003	− 0.002	0.002		− 3.5	4.6	− 2.2
Richer		0.314	0.277	0.294		− 0.015	0.002	− 0.008		15.1	− 4.4	8.7
Richest		0.771	0.746	0.755		− 0.050	0.004	− 0.050		51.1	− 9.1	53.1
Outpatient care utilization												
Concentration index		0.0821	0.0348	0.0347								
Education												
Primary		− 0.187	− 0.196	− 0.228		− 0.005	0.006	− 0.024		− 6.5	17.3	− 68.8
Secondary		0.228	0.143	0.105		0.002	− 0.002	0.005		2.7	− 5.6	13.3
Higher		0.564	0.525	0.530		0.004	0.005	0.000		4.5	14.8	0.4
Wealth index												
Poorer		− 0.486	− 0.526	− 0.485		− 0.010	− 0.026	− 0.005		− 12.1	− 74.8	− 15.1
Middle		− 0.106	− 0.137	− 0.114		− 0.004	− 0.008	− 0.003		− 5.3	− 23.9	− 9.3
Richer		0.314	0.277	0.294		0.029	0.021	0.013		34.7	61.6	36.7
Richest		0.771	0.746	0.755		0.081	0.065	0.057		99.0	187.6	164.8
Inpatient care utilization												
Concentration index		0.0496	0.0625	− 0.0010								
Education												
Primary		− 0.187	− 0.196	− 0.228		− 0.016	− 0.006	− 0.011		− 33.0	− 9.6	113.8
Secondary		0.228	0.143	0.105		0.012	− 0.003	0.004		23.2	− 4.3	− 36.8
Higher		0.564	0.525	0.530		0.014	− 0.005	− 0.001		27.8	− 8.2	5.2
Wealth index												
Poorer		− 0.486	− 0.526	− 0.485		− 0.013	− 0.014	− 0.016		− 27.2	− 22.7	161.9
Middle		− 0.106	− 0.137	− 0.114		− 0.003	− 0.013	− 0.005		− 6.1	− 21.4	50.4
Richer		0.314	0.277	0.294		0.012	0.017	0.022		23.4	27.6	− 222.2
Richest		0.771	0.746	0.755		0.040	0.050	0.029		81.0	80.2	− 291.4

Data for self-reported illness, outpatient care utilization, and inpatient care utilization were not included in the 2003 survey

^a^National Health Insurance Program

^b^Outpatient care utilization and inpatient care utilization

^c^Concentration index

#### Concentration curve

**Fig. 1:**
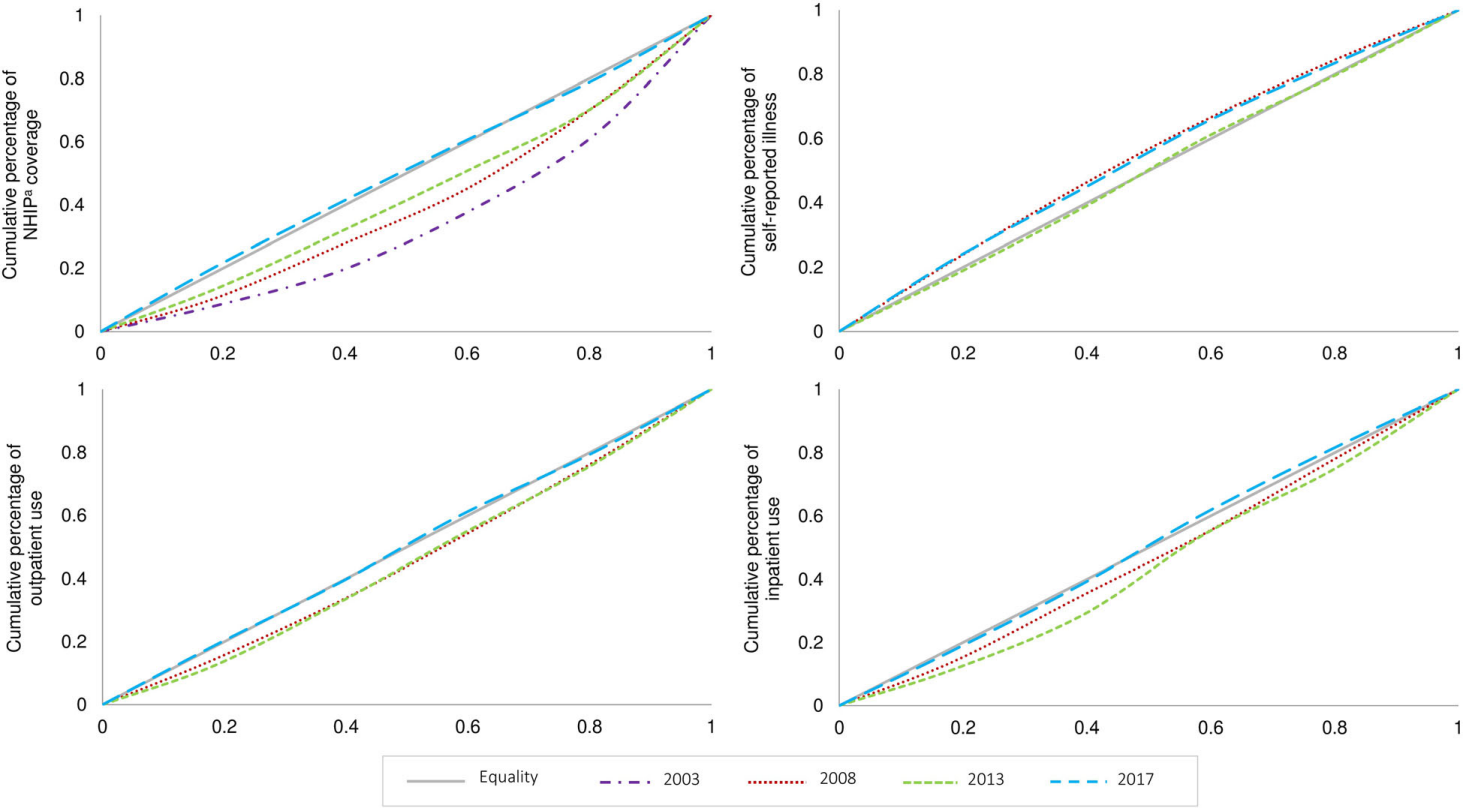
Concentration curves for NHIP^a^ coverage, self-reported illness, and healthcare utilization^b^ according to wealth index (2003-2017). ^a^National Health Insurance Program; ^b^outpatient care utilization and inpatient care utilization

The concentration curve (Fig. 1) was computed to illustrate the cumulative distribution of each health and healthcare utilization variable on the *y*-axis against the cumulative distribution of the population ranked according to wealth, beginning with the poorest and ending with the richest (*x*-axis), graphically. If the health or healthcare utilization outcome was more concentrated among the poor, the concentration curve would lie above the line of equality. The farther the curve is above the 45° line, the more concentrated the health or healthcare utilization variable is among the poor.

#### Decomposition analysis of the concentration index

A regression-based decomposition of the concentration index (Table 3) was performed to explain the contribution of each correlate to the reduction of socio-economic inequalities across the population according to wealth [10]. The regression of the outcome variables was performed based on a linear additive regression of health/healthcare utilization. The mean of the health or healthcare utilization outcome and each of its correlates were estimated, followed by the calculation of the concentration indices for each correlate. To determine the effect of the contribution of each correlate on the outcome, the elasticities of the outcome variables with respect to each correlate evaluated at the mean value of each outcome variable (NHIP coverage, self-reported illness, outpatient care utilization, and inpatient care utilization) were calculated [24]. The contribution of each correlate in the model to the health and healthcare utilization inequality was quantified by calculating the absolute contribution to inequality of each correlate, considering the correlates’ respective concentration indices, followed by the computation of the percentage distribution. Survey weights were used, and all the statistical analyses were performed using Stata 15 [23].

## Results

### Key developments in health policies and laws for older adults

Table 1 presents government policies to expand healthcare utilization and the population coverage of the NHIP among older adults. PhilHealth is a government-owned and operated corporation created in 1995 to implement the NHIP, with the aim of reducing out-of-pocket spending and inequities in health financing [25] and improving access to quality care. Financial assistance provided by the government is limited to the payment of premiums on behalf of disadvantaged groups, termed as “indigent.” Major advancements were implemented towards covering the poorest sector of the population and improving access to quality care, such as the enrollment of poor families, the reduction of hospital fees in government facilities, the allocation of revenues for health, and the expansion of NHIP coverage to include all senior citizens beginning in 2014. The commencement of the *Kalusugang Pangkalahatan*, or the Universal Health Coverage, to address inequities in the health system in 2010 was composed of upgrading facilities under the Health Facilities Enhancement Program (HFEP) to become accredited for Primary Care Benefits beginning in 2012, extending subsidies to the poor and the near poor in 2013 [26], and the mandatory enrollment of senior citizens aged 60 years or older in PhilHealth in 2014. The budget for HFEP reported an increase from PHP 3.25 billion (Philippine peso) in 2010 to PHP 26 billion in 2016 [9]. Republic Act 10645, an act providing mandatory PhilHealth coverage for all senior citizens [27], attempted to provide financial risk protection and access to essential health services by qualifying Filipino citizens who are residents of the Philippines aged 60 years or older and who are not currently covered by any membership category of PhilHealth. By virtue of this Act, a total of 5.8 million older adults were automatically enrolled in PhilHealth in 2015 [9] which later on increased to 6.9 million in 2017 [28].

The premium contributions for recipients of the mandatory membership under the senior citizen category are sourced from revenue from the Sin Tax Law (Republic Act 10351), which assists in the financing of the UHC program [29]. From 2015, the proceeds of the Sin Tax Law were allocated toward the NHIP premiums for both poor and older adults. Overall, 67.5% of older adults included in the senior citizen program of PhilHealth in 2015 reported that they had not had previous coverage. Policies to increase healthcare access through minimizing the social gradient in health were the implementation of the “No Balance Billing” which explicitly forbids government-owned hospitals from charging patients additional fees above what PhilHealth reimburses for case rates, and the “Z Benefit Package” which increases financial risk protection through cost-effective interventions.

### Socio-economic characteristics

Table 2 reports the outcome variables for the four surveys according to socio-economic characteristics among older adults. The rates of NHIP coverage in the four surveys were 9.4% (2003), 35.3% (2008), 57.9% (2013), and 87.6% (2017), showing an increase over the 14-year period. Self-reported illness increased to 28.3% in 2017 from 20.5% in 2008. Outpatient care utilization and inpatient care utilization increased from 13.3 in 2008 to 18.7% in 2017 and from 8.8 in 2008 to 12.4% in 2017, respectively.

Wealth is significantly associated with self-reported illness in three of the surveys (2008: *P* ≤ 0.001; 2013: *P* = 0.02; 2017: *P* ≤ 0.001), with the poorest reporting a higher occurrence of disease. Self-reported illness shows positive association with NHIP coverage in 2013 and 2017. Wealth is also significantly associated with outpatient care utilization from 2008 to 2017, with richer individuals reporting higher utilization. Inpatient care utilization from 2013 to 2017 is significantly associated with wealth, but a trend was not observed among wealth quintiles.

### Inequalities in NHIP coverage, self-reported illness, outpatient care utilization, and inpatient care utilization

Table 3 presents the concentration indexes, and Fig. 1 illustrates the concentration curves for NHIP coverage, self-reported illness, outpatient care utilization, and inpatient care utilization. The concentration index for NHIP coverage was 0.3000 in 2003 and 0.0247 in 2017. Figure 1 illustrates a pro-rich inequality in NHIP coverage, which indicates that NHIP coverage is more prevalent among wealthier older adults. Self-reported illness shows a pro-poor inequality for 2008 to 2017, with a concentration index of − 0.0972 and − 0.0965, respectively. This demonstrates that the greater proportion of older Filipino adults reporting the presence of illness is poor. Furthermore, a decrease in the concentration index of self-reported illness was observed between 2008 and 2013, with a value of − 0.0972 and − 0.0474, respectively.

Outpatient care utilization shows a pro-rich inequality, with a decrease in the concentration index from 0.0821 (2008) to 0.0347 (2017); this change moved the concentration curve to a position almost equivalent to the line of equity. For inpatient care utilization, a positive concentration index of 0.0496 in 2008 increased to 0.0625 in 2013, both denoting a pro-rich inequality. Following 2013, the concentration index shifted to a negative value of − 0.0010 in 2017, revealing a pro-poor inequality, indicating that poorer older adults use inpatient care more than the wealthier groups.

### Decomposition analysis of socio-economic inequalities

Table 3 presents the contributions of education and wealth to socio-economic inequalities. The contributions of older adults belonging to a wealthier group were 48.3% (2003), 66.3% (2008), 59.5% (2013), and 75.5% (2017), explaining the pro-rich inequality in NHIP coverage. For self-reported illness, the contribution of urban residence decreased to 5.3% (2017) from 25.4% (2008) and 45.9% (2013). For outpatient care utilization, the percentage contribution of having a secondary education or higher increases from 7.2 in 2008 to 13.7% in 2017. The wealth index shows a decrease in its contribution to the concentration index of inpatient care utilization from 2008 to 2017.

## Discussion

Less than half of older adults living in the Philippines were covered by the NHIP between 2003 (9.4%) and 2008 (35.3%); an increase was observed in 2013 (57.9%), and the majority of older adults were covered in 2017 (87.6%) after the implementation of the NHIP coverage expansion among older adults. These findings showed an increase in NHIP coverage among poorer older adults from 2003 to 2017, despite the persistence of a pro-rich inequality (Fig. 1). Laws and policies have been mandated to increase subsidized NHIP coverage among indigents and older adults, with participation from local government units (LGU) and the DOH.

### Equity in NHIP coverage

Our study showed a decline in socio-economic inequalities in NHIP coverage after the mandatory NHIP coverage for older adults, as indicated by the increase in coverage between 2003 and 2017. Between 2003 and 2008, several laws were enacted, such as the granting of additional benefits and privileges to older adults, subsidies for NHIP premium contributions for indigent members by LGUs and the national government, the enrollment of an additional 4.2 million poor families into the NHIP, and the allocation of 2.5% of government revenues from taxes on “sin” products to the NHIP, which contributed to a substantial increase in new coverage [30].

Further initiatives were enacted between 2010 and 2014, such as the implementation of Universal Health Care or *Kalusugang Pangkalahatan* to address inequity in the health system, the “No Balance Billing” Policy protecting the poor from paying fees in excess of the NHIP coverage at government facilities, the Sin Tax Law that subsidizes NHIP coverage premiums, the introduction of Z-Benefits [2], which aims to cover the expenses of costly procedures, and the introduction of the Expanded Senior’s Citizens’ Act of 2010 stipulating the automatic NHIP coverage of all indigent older adults and later on amended to include the mandatory enrollment of all adults aged 60 years or older into the NHIP in 2014 [4]. The DOH reported that by 2015, a total of 5.8 million older adults had been automatically enrolled in PhilHealth, and a Sin Tax Law incremental revenue of PHP 31.26 billion was allocated in 2016 to cover the 1-year NHIP premium subsidies of indigents and older adults not covered by the NHIP, representing a 248% increase from the 2013 DOH budget level [31]. So far, no data has been reported evaluating whether the goals of these laws and initiatives have been achieved, but we can speculate that the reduction in inequalities in NHIP coverage is related to the expansion of NHIP coverage for older adults. The government’s initiative to expand NHIP coverage was sequentially followed by older adults gaining easier access to NHIP coverage regardless of their wealth status; however, causal inferences are unascertained in the present study.

With advancements in NHIP coverage expansion and increased allocation for NHIP premium subsidies, out of pocket spending remains to gather the majority portion of total health expenditure, covering 53.7% in 2014 [6]. Utilization of healthcare services largely depends on the household’s ability to pay, with the share of PhilHealth in total health expenditure at only 14% [2].

### Health inequalities

Following the expansion in NHIP coverage among older adults in 2014, decrease in healthcare utilization inequalities was observed. The negative concentration index for self-reported illness in 2008, 2013, and 2017 indicated a concentration among the poorer population. Health policy reforms on increasing health insurance coverage may have contributed to the increase access to healthcare services such as diagnosis and treatment [32] leading to a better health outcome [33]. The financial risk protection afforded by the revenue for health from the Sin Tax Law expanded the DOH’s budget and enabled greater medical assistance, with a figure of 24,009 indigents benefiting in 2013 and 363,900 benefiting in 2016 [31].

The concentration of self-reported illness among older adults with lower income may also be explained by a decreased means of affording healthcare, leading to an inability to respond to healthcare needs, in contrast to richer individuals with the ability to purchase healthcare services [34, 35]. Pathways linking income inequality and health have been reported in past years, suggesting that income poverty is a risk factor for premature mortality and increased morbidity [36, 37]. A study done in the USA suggested that poverty is considered to be the primary influence in access to healthcare among the elderly [38].

### Access to healthcare

The concentration index for inpatient care utilization shifted from being pro-rich between 2008 and 2013 to being pro-poor inequality in 2017, 3 years after the initiation of the expansion of NHIP coverage to include older adults. Inequalities were reduced as evidenced by the concentration indexes moving towards a score of 0 and the concentration curves situated close to or over the line of equity. The initiation of laws, such as providing discounts, free healthcare utilization and medicines in government hospitals, and the mandatory enrollment of older adults into the NHIP, was sequentially followed by a narrowing of socio-economic inequalities and an increase in the utilization of healthcare services among older adults.

Risk factors such as wealth and education are important contributors to health utilization inequalities based on decomposition analyses [12, 39]. Various health conditions, an older age, and the availability of health insurance play important roles in enabling the use of certain services more than others [20]. Studies in LMICs have reported that wealth status or economic factors are associated with healthcare utilization [19, 20], similar to the results in high-income countries [38]. Furthermore, the present findings are consistent with those of a study done in China showing a pro-rich inequality in inpatient utilization, indicating that a disproportionate share of inpatient resources are utilized by wealthier individuals with lower health needs [13]. An increase in socio-economic inequality of inpatient utilization was observed in 2013 compared to 2008, considering the policies on increasing financial risk protection put in place (Table 1). PhilHealth benefit utilization remained low despite increase in NHIP coverage. Low PhilHealth utilization rates may be explained by the avoidance of the poor to seek care in hospitals because of catastrophic medical expenses that hospitalization requires, or scarcity of health workforce and facilities capable to provide quality care in geographically isolated areas, leading to unattainable healthcare services [40].

Meanwhile, a pro-poor inequality was observed for inpatient utilization in 2017, which can be explained by individuals belonging to the poorer quintiles reporting a greater proportion of inpatient care use, compared with those in the wealthier cohort, following the expansion of NHIP coverage among older adults, despite the strong pro-rich inequality in healthcare use reported in previous studies [12, 13]. This change may be attributable to new laws and policies mandating free medical and dental services in 2010 and the mandatory inclusion of individuals aged 60 years or older in the “No Balance Billing” policy of PhilHealth in 2015 [41, 42]. Furthermore, the increased budget allocation for the HFEP, which provides primary care benefits, might have contributed to easier access to quality healthcare for older Filipino adults.

Data from the year 2017 showed a higher utilization of both outpatient and inpatient healthcare services, compared with previous years. Despite having a lesser need for healthcare services, richer individuals generally utilize more health services than poorer individuals, suggesting that income is the principal determinant of the pro-rich inequality in healthcare utilization [13]. Therefore, wealthier older adults pay for more services, regardless of need. Research examining healthcare utilization among older adults with cardiovascular diseases reported that older age, gender, higher household wealth status, higher education, and health insurance were associated with outpatient care utilization [19, 20, 39], while only age and health insurance were associated with inpatient care utilization [19]. In contrast, a study done in Indonesia revealed that an older adult’s insurance status was associated with a higher use of inpatient services and that the use of outpatient services varied among regions [43], which may be explained by the partial resolution of financial barriers to care and differences in healthcare coverage/availability of service providers, respectively. A report that health insurance is a strong contributor to inpatient care utilization among older adults [19] supports our findings of a pro-poor inequality for inpatient care utilization following NHIP coverage expansion. Aside from minimizing socio-economic inequalities in healthcare utilization, achieving good health outcomes is of equal importance. Furthermore, decrease in outpatient care utilization inequality was also observed but remained pro-rich before and after the NHIP coverage expansion policy. Political commitment encompassing experimental verification, risk analysis, and sustained citizen education [44] must be ensured not only to expand healthcare coverage but also to guarantee affordability and the availability of various benefit packages [45].

## Strengths and limitations

The methodological strengths of this study include the calculation of the concentration index and the decomposition analysis of *C*. The concentration index includes all individuals in its calculation and is sensitive to changes in the distribution of health and healthcare utilization within the population across different socio-economic categories [46], while decomposition analysis of the concentration index was applied to determine the contributions of socio-economic variables and individual characteristics on health and healthcare utilization inequalities in nationally representative samples. Next, the study analyzed the change in socio-economic inequalities in NHIP coverage, health, and healthcare utilization among older adults in the Philippines before and after the NHIP coverage expansion in the older adult population. However, the present study has some potential limitations, and caution is needed in interpreting trends in health and healthcare utilization based on the DHS wealth index, considering that the index shows a relative position measured using a composite economic status indicator among the participants of a particular year and country, which prevents the wealth index scores for different years from being compared [47]. Furthermore, the cross-sectional data used cannot be employed to infer causality. The DHS performed in the Philippines was not designed to fill a gap in research on ageing specifically, which limited the variables that could be used to explain inequalities in health among older adults accurately. The reporting of illness is subjective and cannot be used to determine severity precisely. NCD, CD, and injuries also present differences in medical-seeking behavior, depending on the disease severity. Likewise, healthcare utilization is also subjective; therefore, the utilization of inpatient services, for example, does not necessarily mean that those utilizing the services are ill or that their health conditions require hospitalization. DHS data does not distinguish between the use of private health facilities accredited by PhilHealth and those not accredited at the time of interview; therefore, the data used is unable to ascertain that increase access to healthcare is solely contributed by NHIP. Furthermore, the self-reporting of healthcare use cannot be used to determine whether those who utilized outpatient services also utilized inpatient services as recommended by a healthcare professional. Identifying mortality through using death information in health insurance claims databases [48] may be considered to assess inequalities in healthcare use in future researches. Lastly, it is worth noting that despite the resulting massive coverage of older adults in the NHIP, this populist policy offering free healthcare may cause an overuse of services leading to a heavy financial burden on the Philippine health system, as experienced in Japan, wherein challenges such as financial sustainability and equity remain, and actions to reduce healthcare expenditures have led to the abolishment of free healthcare for older adults, the requirement of a 10% out-of-pocket fee, and an increase in the age of eligibility [5].

## Conclusions

Overall, the results presented an increase in the percentage of and a reduction in socio-economic inequalities in NHIP coverage, health, and healthcare utilization among older adults, especially those belonging to lower-income groups. The sequential outcomes following instituted health and social policies showed that increased investment in NHIP coverage expansion led not only to a decline in health and healthcare utilization inequalities but also enabled equal opportunities to access health services, which is a major component leading towards UHC.

## Data Availability

The datasets analyzed during the current study are available in the DHS data repository: https://www.dhsprogram.com/.

## Abbreviations

NHIP: National Health Insurance Program
LMIC: Low- to middle-income countries
PhilHealth: Philippine Health Corporation
DOH: Department of Health
USD: US dollars
UHC: Universal Health Coverage
PDHS: Philippine National Demographic and Health Survey
NCD: Non-communicable diseases
CD: Communicable diseases
*C*: Concentration index
HFEP: Health Facilities Enhancement Program
PHP: Philippine peso
LGU: Local government units
